# Changes of liver transcriptome profiles following oxidative stress in streptozotocin-induced diabetes in mice

**DOI:** 10.7717/peerj.8983

**Published:** 2020-05-27

**Authors:** Shuren Guo, Xiaohuan Mao, Yunmeng Yan, Yan Zhang, Liang Ming

**Affiliations:** 1 Department of Clinical Laboratory, The First Affiliated Hospital of Zhengzhou University, Zhengzhou, Henan, People’s Republic of China; 2 Department of Clinical Laboratory, Henan Provincial People’s Hospital, People’s Hospital of Zhengzhou University, Zhengzhou, Henan, People’s Republic of China

**Keywords:** Oxidative-stress, STZ, Differentially expressed RNAs, Metabolism, Liver

## Abstract

**Background:**

Oxidative-stress (OS) was causal in the development of cell dysfunction and insulin resistance. Streptozotocin (STZ) was an alkylation agent that increased reactive oxygen species (ROS) levels. Here we aimed to explore the oxidative-stress and related RNAs in the liver of STZ-induced diabetic mice.

**Methods:**

RNA-sequencing was performed using liver tissues from STZ induced diabetic mice and controls. Pathway and Gene Ontology (GO) analyses were utilized to annotate the target genes. The differentially expressed RNAs involved in the peroxisome pathway were validated by qRT-PCR. The glucose metabolite and OS markers were measured in the normal control (NC) and STZ-induced diabetic mellitus (DM) group.

**Results:**

The levels of serum Fasting insulin, HbA1c, Malondialdehyde (MDA) and 8-iso-prostaglandin F2*α* (8-iso-PGF2*α*) were significant higher in DM groups than NC group, while SOD activity decreased significantly in DM groups. We found 416 lncRNAs and 910 mRNAs were differentially expressed in the STZ-induced diabetic mice compared to the control group. OS associated RNAs were differentially expressed in the liver of STZ-induced diabetic mice.

**Conclusion:**

This study confirmed that the OS was increased in the STZ-induced DM mice as evidenced by the increase of lipid peroxidation product MDA and 8-iso-PGF2*α*, identified aberrantly expressed lncRNAs and mRNAs in STZ-induced diabetic mice.

## Introduction

Diabetes mellitus, characterized by a rise in plasma glucose levels, is one of the most common chronic metabolic diseases in the world. The liver is an important insulin target organ, regulating glucose and lipid metabolism, and is also a crucial place for insulin resistance (DeFronzo, 2004; Tan & Cheah, 1990). The formation of reactive oxygen species (ROS) is an inevitable byproduct of metabolism. Oxidative stress (OS) is induced by an abundance of ROS or failure in the anti-oxidative machinery. OS played a key role in pathological processes observed in T2DM (Fernandes et al., 2016; Henriksen, Diamond-Stanic & Marchionne, 2011). Recent studies indicated that oxidative stress was also causal in the development of cell dysfunction and insulin resistance (Leahy, 2005; Nahdi, John & Raza, 2017; Raza & John, 2012).

Streptozotocin (STZ) was an alkylation agent that increased ROS levels and damaged the antioxidant system in the islet cells during the induction of experimental diabetes model. The damaged antioxidant system resulted in the rupture of DNA chain and further led to beta cell necrosis (Lao-ong et al., 2012; Papaccio et al., 1991). In the meantime, the metabolism of STZ by the liver microsomal P450 enzyme system could produce toxic electrophilic substances, such as acrolein, which can bind with proteins, nucleic acids and lipids, and led to changes in the activity of important functional enzymes, thus causing hepatic oxidative stress injury (Ahn, Yun & Oh, 2006). In addition to the direct chemical damage, the OS caused by STZ also induced a rapid and transient global transcription change. It has been verified in fibroblast cells that pausing of RNA polymerase II (PolII) in both directions, at specific promoters occurred within 30 min of the OS. PolII pausing could lead to the generation of thousands of long noncoding RNAs (lncRNAs) with promoter-associated antisense lncRNAs transcripts (Giannakakis et al., 2015).

lncRNAs are larger than 200 nucleotides in length (Mattick & Makunin, 2006), and are widely expressed across the genome. lncRNAs are master regulators in gene regulation and cellular function as signals, molecular decoys, or scaffolds.

Recent studies demonstrated that lncRNAs were important players in diabetes and its complications (Leung & Natarajan, 2018). In spite of the detail reports of lncRNAs changes in the fibroblast cells upon OS, the whole transcription profile of lncRNAs in the liver cells induced by the STZ was not completely understood. In our study, we sequenced the whole transcription profiles in the STZ-induced DM mice liver cells, aimed to explore the differentially expressed OS-related RNAs, assess their physiological effects and correlate them to the altered hepatic physiology during diabetes.

## Material and Methods

### Animals

Specific pathogen-free male C57BL/6 mice weighing 20–22 g were purchased from the Organ transplantation center, Tongji hospital affiliated with Tongji Medical College, Huazhong University of Science and Technology. The Zhengzhou University Animal Care and Use Committee approved all animal experiments (the approval number 2017051805), which were performed in accordance with ‘Animal Research: Reporting In Vivo Experiments’ (ARRIVE) guidelines. After 12 h of fasting, mice received one intra-peritoneal injection of 130mg/kg streptozotocin (STZ, Sigma, St. Louis, MO, USA) solution in 0.05 M citrate buffer (pH 4.5) to induce diabetes (DM, *n* = 20) (Deeds et al., 2011; Huang Xin & Cao, 2007; Mallek et al., 2018). Normal Control group were injected with citrate buffer (NC, *n* = 10). Blood glucose (BG) was measured to confirm diabetes, which was defined as glycemia higher than 16.7 mmol/L.

After injection, the mice continued to receive a high-fat diet for another 2 weeks. During the 2 weeks, three animals in the DM group died. Blood glucose level of mice was tested from the tip of the tail. Four weeks after the injection, the mice were euthanized by intraperitoneal injection of 250 mg/kg body weight pentobarbital (Sigma, P3761, under sterile conditions) to harvest liver samples, and blood was collected from the orbital vein to measure serum biochemical markers.

### Biochemical marker measurement

Then fast plasma glucose and insulin level of diabetic mice (*n* = 17) and normal control mice (*n* = 10) were tested and compared to each other. Serum glucose, insulin levels and total SOD activity were tested by the Automatic biochemical analyzer (cobas 8000 series) using Roche regents according to the manufacture instruction. MDA formed from the breakdown of polyunsaturated fatty acids serves as a convenient index for the determination of the extent of peroxidation reaction. MDA, a product of lipid peroxidation, reacts with thiobarbituric acid to give a pink-colored product, having a maximum absorption at 535 nm (Nair & Nair, 2017). 8-iso-PGF2*α* was determined with a competitive enzyme-linked immunosorbent assay (ELISA) (Stressgen Biotechnologies Inc., San Diego, CA, USA). HbA1c was detected by High Performance Liquid Chromatography with Borate Affinity Chromatography.

### Total RNA extraction and purification

Total RNA from liver tissues of normal and diabetic mice was isolated using the NEB Next Ultra Directional RNA LibraryPrep Kit for Illumina (NEB, Ispawich, USA) and quantified using Agilent 2100 RNA Nano 6000 Assay Kit (Agilent Technologies, CA, USA). 3 µg of total RNA was used for sequencing preparation using NEB Next Ultra Directional RNA LibraryPrep Kit for Illumina (NEB, Ispawich, USA) kit along with Ribo-Zero Gold rRNA (Illumina Inc., CA, USA) to remove rRNA according to the previous study (Zhang et al., 2014). The resulting libraries were sequenced on a HiSeq 2000 (Illumina Inc., CA, USA) instrument that generated paired-end reads of 100 nucleotides.

### Illumina HiSeq2000 analysis

RNA extracted from the liver tissues of three control mice were pooled together for sequencing. The sequencing reads were obtained from control pools and STZ-induced diabetic mice (*n* = 3). Raw sequencing reads were further processed with Perl scripts to exclude the adaptor-polluted reads, low-quality reads and reads with the number of N bases accounting for more than 5%. Q30 statistics was performed to test the data quantity and quality. And Clean Data were mapped to the reference genome (http://www.ensembl.org/index.html) using HISAT2 (http://ccb.jhu.edu/software/hisat2/index.shtml) (Kim, Langmead & Salzberg, 2015). The liver transcriptome was reconstructed from all of the RNA-seq datasets using StringTie 1.3.2.d (http://ccb.jhu.edu/software/stringtie/). DESeq (http://www.bioconductor.org/packages/release/bioc/html/DESeq. html) was used for differential expression analysis between diabetic and normal mice liver transcriptomes. Differentially expressed genes were identified based on threshold changes of ≥2-fold or ≤-2-fold and q values ≤ 0.05. The data were normalized and hierarchically clustered with R software 3.1.1. The potential function of the differentially expressed genes was analyzed by Gene ontology and Pathway analysis. The enriched genes in Kyoto Encyclopedia of Genes and Genomes (KEGG) were calculated by hypergeometric distribution.

### Quantitative real-time PCR (qRT-PCR) and statistical analysis

Total RNA was extracted using the RNeasy kit (Qiagen, Inc., Valencia, CA, USA) according to the manufacturer’s instructions, and 2 µg purified RNA was reverse transcribed into cDNA (37 °C for 15 min, followed by 85 °C for 5 s using RT kit; Fermentas; Thermo Fisher Scientific, Inc.). Primers for qRT-PCR were designed based on the sequences from ensembl (http://asia.ensembl.org/index.html). qRT-PCR was performed using the ABI 7500 Real-Time PCR using a QuantiTect SYBR Green PCR kit (Qiagen, Inc.). The qRT-PCR cycle was pre-denaturation at 95 °C for 3 min, followed by 35 cycles of denaturation at 95 °C for 5 s and annealing at 60 °C for 30 s, and a final analysis from 60−95 °C. qRT-PCR results were quantified using the 2^−ΔΔ*ct*^ method. *β*-*actin* was chosen as a reference gene. All the gene expression levels were normalized to *β*-*actin* measured in parallel.

qRT-PCR assays were performed in triplicate and the data represented the means of three experiments. All data were represented as mean  ± standard deviation. Comparison between groups was performed using the independent sample Student t test with *P* < 0.05 as the criterion for statistical significance. All analyses were done using SPSS statistics (version 17.0) and GraphPad Prism (version 7).

## Results

### Biochemical parameters of two group mice after 4 weeks of injection

**Figure 1:**
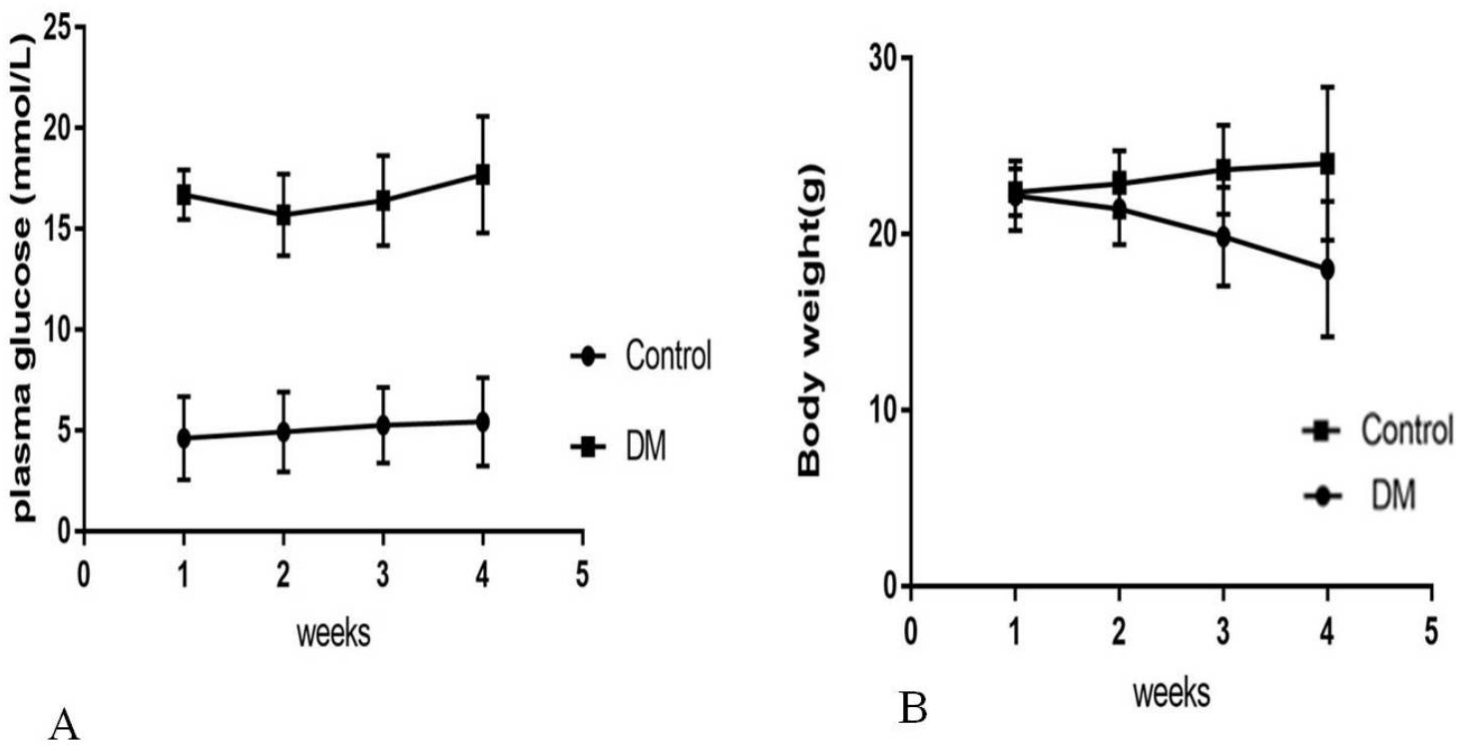
Biochemical parameters in control mice and STZ-induced diabetic mice over 4 weeks. (A) Plasma concentration of fasting blood glucose. (B) Body weight.

Four weeks after the injection, control mice were weighing 24.02  ± 4.35 g with blood glucose levels 5.4  ± 0.82 mmol/L, and diabetic mice were weighing 18.02  ± 3.86 g with blood glucose levels 17.4  ± 3.28 mmol/L (Fig. 1). Four weeks after STZ injection, the distribution of biochemical parameters in serum of control and diabetic groups was shown in Table 1. The levels of serum Fasting insulin, HbA1c, MDA and 8-iso-PGF2*α* were significant higher in DM groups than NC group, while SOD activity decreased significantly in DM groups. Fast insulin level and Body weight were significant lower in DM groups compared to NC group.

### RNA sequence data generation and quality control

We observed that administration of streptozotocin caused a significant increase in plasmatic glucose and a decrease in insulin levels. Whole transcriptome RNA sequencing of liver tissue was performed to identify differentially expressed RNAs related to the OS. RNA extracted from the liver tissues of three control mice were pooled together as control group, while the RNA samples from liver tissues of three diabetic mice were sequenced separately as diabetic group. We obtained a total of 6.0746 × 10^8^ raw reads (Table S1, Fig. S1A). The raw data is available at NCBI (accession number PRJNA562053). The Q30 Bases Rate was more than 95% by Q30 statistics (Fig. S1B). We found that a large fraction (median percentage, 68.86%) of the sequence was overlapped by exon regions and that only a small fraction (median percentage, 3.305%) was mapped to the intergenic region (Fig. S1C). Interestingly, long intergenic non-coding RNAs (lincRNA) always located in these areas.

**Table 1:** Distribution of various biochemical parameters of control (*n* = 10) and diabetic groups (*n* = 17) four weeks after STZ injection (x ± s).

Groups	Diabetic groups	Normal groups	*P*
Weight (kg)	18.02 ± 3.86	20.4 ± 3.28	<0.001
Fast Blood Glucose (mmol/L)	24.02 ± 4.35	5.4 ± 0.82	<0.001
Fast Insulin level (mIU/L)	13.45 ± 8.25	31.45 ± 3.32	<0.001
MDA (nmol/L)	1.90 ± 0.78	0.90 ± 0.28	<0.001
Total SOD (U/ml)	0.80 ± 0.3	1.10 ± 0.2	<0.001
Cytochrome c((pmol/L)	3.2 ± 0.58	1.70 ± 0.6	0.004
8-iso-PGF2*α* (ng/ml)	2.57 ± 0.83	1.23 ± 0.48	<0.001

### Differential expression analysis of liver transcriptomes

**Figure 2:**
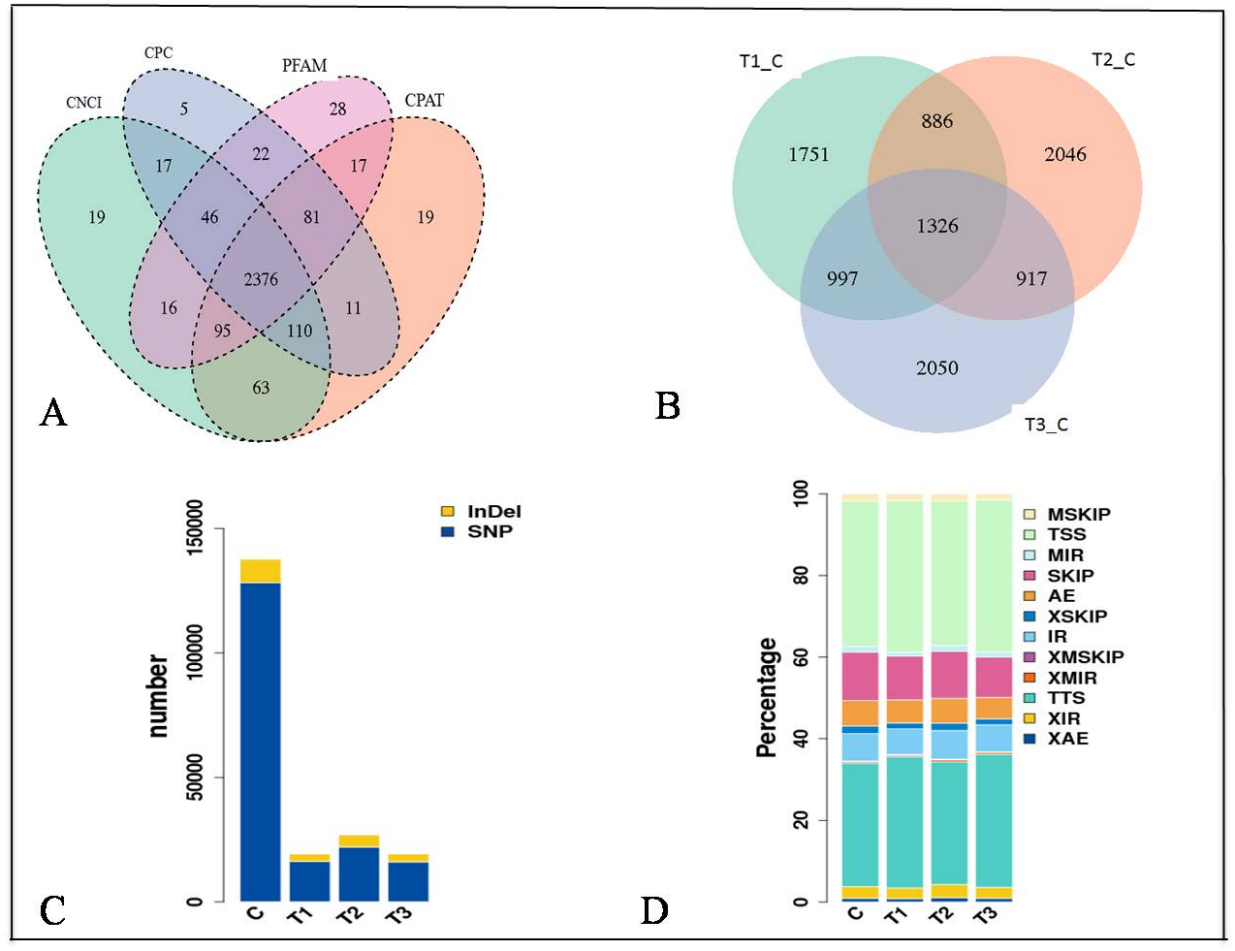
Characteristic of RNA-sequence data. (A) Venn diagram of Novel lncRNAs identified by 4 methods, (B) Differentially expressed lncRNAs, (C) Variation statistics, (D) Alternative splice statistics, SKIP, Skipped Exon; SKIP, Multi-exon SKIP; IR, Intron Retention; MIR, Multi-IR; AE, Alternative Exon Ends; TSS, Transcription Start Site; TTS, Transcription Terminal Site; XSKIP, Approximate SKIP; XMSKIP, Multi-exon SKIP; XIR, Approximate IR; XMIR, Approximate MIR; XAE, Approximate AE.

The expression of genes was quantified as Fragments Per Kilobase of transcript per Million mapped reads (FPKM) values (Figs. S1D and S1E). The distribution of the gene expression pattern was similar between the diabetic and normal control mice, only a small fraction of genes were differentially expressed. We identified a total of 2376 novel lncRNAs (Figs. 2A, 2B) and 1326 differentially expressed genes (Table S2). Of which there were 287 up regulated mRNAs and 623 down regulated mRNAs, 161 up regulated lncRNAs and 255 down regulated lncRNAs in the STZ-induced diabetic mice compared to the normal control mice (Table S2). The average percentages of SNP variations in the control and DM group were 93% and 83% (Fig. 2C). The alternative splice statistics showed that the splice occurred mainly in Transcription Start Site (TSS) and Transcription Terminal Site (TTS) (Fig. 2D).

**Figure 3:**
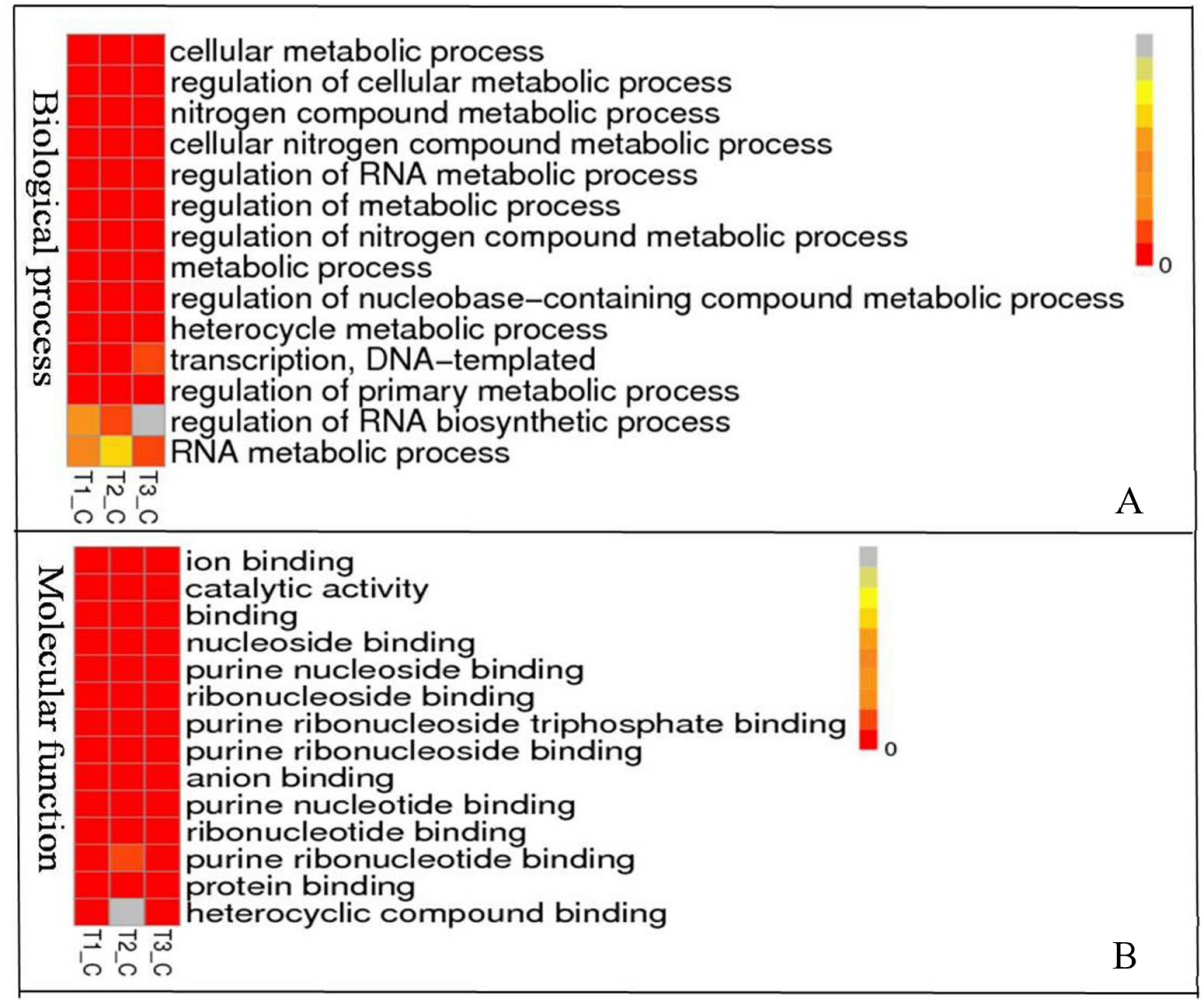
Representation Go terms of differentially expressed genes in the STZ-induced DM mice liver. The vertical ordinate represents Go term, the horizontal ordinate represents sample name. The different color represents the enrichment degree. (A) Biological process enrichment of dysregulated lncRNAs. (B) Molecular function enrichment of dysregulated lncRNAs.

To understand the biological pathways and functions altered in STZ-induced diabetic mouse liver, gene ontology and pathway enrichment analysis were utilized to annotate the target genes. GO analyses found that the dysregulated lncRNAs associated with diabetes mellitus were associated with regulation of apoptotic signaling pathway, negative regulation of transcription from RNA polymerase II promoter, fatty acid catabolic and oxidation process, protein modification and localization (Fig. 3).

**Figure 4:**
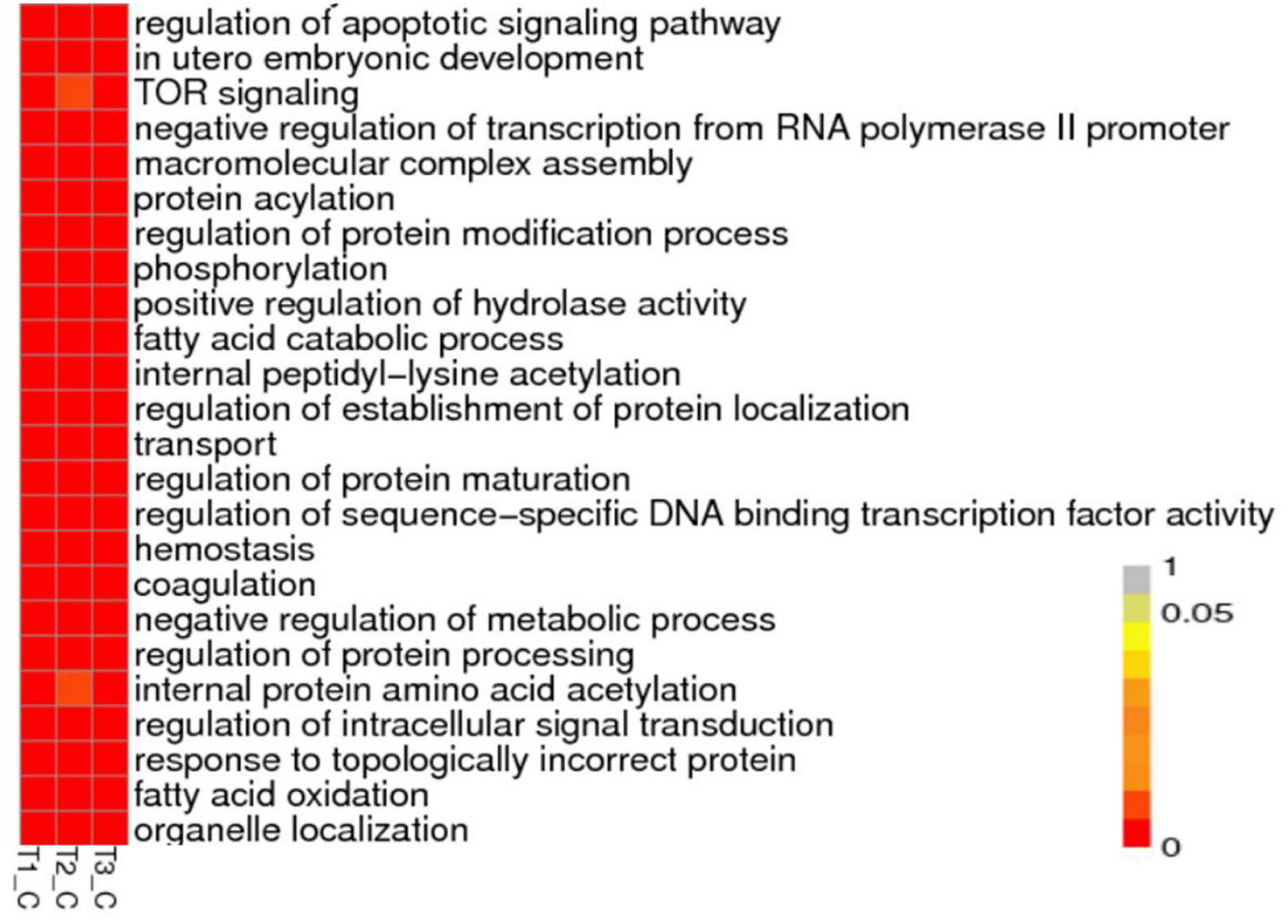
Representation pathway terms of differentially expressed genes in the STZ-induced DM mice liver. The vertical ordinate represents the KEGG Go term, the horizontal ordinate represents sample name. The different color represents the enrichment degree.

KEGG (Kyoto Encyclopedia of Genes and Genomes, http://www.kegg.jp/) pathway analysis showed the dysregulated lncRNAs mainly involved in four categories (Fig. 4, Table 2). The first category is inflammation, such as hepatitis B, epstein-Barr virus infection, protein processing in endoplasmic reticulum, lysosome and Toll-like receptor signaling pathway. The other one is peroxisome, which is closely related to OS. Another category is cell cycle and cell apoptosis, including eurotrophin signaling pathway, TNF signaling pathway and ubiquitin mediated proteolysis. The fourth category is insulin signaling pathway.

**Table 2:** Effects of STZ on hepatic gene expression.

KEGG pathway/Gene	Gene name		Type	Fold change
Toll-like receptor signaling pathway				
ENSMUSG00000025498|K09447	Irf7	Interferon regulatory factor 7	mRNA	2.58
ENSMUSG00000085667|K00922	Gm12992	PREDICTED: phosphatidylinositol 4,5-bisphosphate 3-kinase catalytic subunit beta isoform isoform X2 [Mus musculus]	processed_transcript	2.03
ENSMUSG00000021250|K04379	FOS	Proto-oncogene protein c-fos	mRNA	0.54
ENSMUSG00000027398|K04519	IL1B	Interleukin 1 beta	mRNA	0.48
ENSMUSG00000051439|K04391	CD14	Monocyte differentiation antigen	mRNA	0.49
ENSMUSG00000020901|K02649	PIK3R1_2_3	Phosphoinositide-3-kinase regulatory subunit alpha/beta/delta	mRNA	2.19
ENSMUSG00000028111|K01371	CTSK	Cathepsin K	0.46
ENSMUSG00000112163|K04427	MAP3K7, TAK1	Processed_pseudogene	Processed_pseudogene	0.16
Epstein-Barr virus infection				
ENSMUSG00000085995|K10591|K09391	Gm2788	Predicted gene 2788	ncRNA	2.57
ENSMUSG00000105987|K10591	AI506816	Expressed sequence AI506816	ncRNA	2.37
ENSMUSG00000004951|K04455	Hspb1	Heat shock protein 1	mRNA	2.01
ENSMUSG00000019942|K02087	Cdk1	Cyclin-dependent kinase 1	mRNA	3.04
ENSMUSG00000073406|K06751	H2-Bl	Histocompatibility 2	mRNA	4.29
ENSMUSG00000085667|K00922	Gm12992	Predicted gene 12992	ncRNA	2.64
ENSMUSG00000112879|K03020	AC158802.2		Processed_pseudogene	4.41
ENSMUSG00000030724|K06465	Cd19	CD19 antigen	mRNA	2.25
ENSMUSG00000038086|K09543	Hspb2	Heat shock protein 2	mRNA	5.11
ENSMUSG00000113137|K03016	AC157515.2		Processed_pseudogene	5.39
ENSMUSG00000078915|K04455	Hsp25-ps1	Heat shock protein beta-1	mRNA	2.15
ENSMUSG00000020901|K02649	Pik3r5	Phosphoinositide-3-kinase regulatory subunit 5	mRNA	0.41
ENSMUSG00000079507|K06751	H2-Q1	Histocompatibility 2, Q region locus 1	mRNA	0.48
ENSMUSG00000112163|K04427	AC158606.2		Processed_pseudogene	0.25
ENSMUSG00000092243|K06751	Gm7030	Predicted gene 7030	ncRNA	0.18
ENSMUSG00000073405|K06751	H2-T-ps		Unprocessed_pseudogene	0.47
ENSMUSG00000075042|K11838	4930431P03Rik	RIKEN cDNA 4930431P03 gene	Processed_transcript	0.09
Hepatitis B				
ENSMUSG00000025498|K09447	Irf7	Interferon regulatory factor 7	mRNA	2.58
ENSMUSG00000070348|K04503	Ccnd1	Cyclin D1	mRNA	2.74
ENSMUSG00000018983|K09389	E2f2	E2F transcription factor 2	mRNA	2.09
ENSMUSG00000081158|K02089	Gm13521		Processed_pseudogene	7.02
ENSMUSG00000097327|K02091	E030030I06Rik	RIKEN cDNA E030030I06 gene	mRNA	2.43
ENSMUSG00000081121|K04364	Gm12791		Processed_pseudogene	8.38
ENSMUSG00000085667|K00922	Gm12992	Predicted gene 12992	ncRNA	2.64
ENSMUSG00000021250|K04379	Fos	FBJ osteosarcoma oncogene	mRNA	0.23
ENSMUSG00000020901|K02649	Pik3r5	Phosphoinositide-3-kinase regulatory subunit 5	mRNA	0.41
Apoptosis				
ENSMUSG00000085667|K00922	Gm12992	Predicted gene 12992	ncRNA	2.64
ENSMUSG00000026072|K04386	Il1r1	Interleukin 1 receptor, type I	mRNA	0.50
ENSMUSG00000027398|K04519	Il1b	Interleukin 1 beta	mRNA	0.31
ENSMUSG00000020901|K02649	Pik3r5	Phosphoinositide-3-kinase regulatory subunit 5	mRNA	0.41
ENSMUSG00000002997|K04739	Prkar2b	Protein kinase, cAMP dependent regulatory, type II beta	mRNA	0.48
Protein processing in endoplasmic reticulum				
ENSMUSG00000057789|K14021	Bak1	BCL2-antagonist/killer 1	mRNA	2.12
ENSMUSG00000083261|K09502	Gm7816		Processed_pseudogene	2.10
ENSMUSG00000007033|K03283	Hspa1l	Heat shock protein 1-like	mRNA	2.55
ENSMUSG00000100615|K04079	Gm5511		Processed_pseudogene	7.36
ENSMUSG00000009092|K13989	Derl3	Der1-like domain family, member 3	mRNA	0.42
ENSMUSG00000090197|K09502	Dnaja1-ps		Processed_pseudogene	0.22
Peroxisome				
ENSMUSG00000031278|K01897	Acsl4	acyl-CoA synthetase long-chain family member 4	mRNA	2.90
ENSMUSG00000020333|K01897	Acsl6	acyl-CoA synthetase long-chain family member 6	mRNA	3.57
ENSMUSG00000007908|K01640	Hmgcll1	3-hydroxymethyl-3-methylglutaryl-Coenzyme A lyase-like 1	mRNA	2.12
ENSMUSG00000055782|K05676	Abcd2	ATP-binding cassette, sub-family D (ALD), member 2	mRNA	0.09
ENSMUSG00000027870|K11517	Hao2	Hydroxyacid oxidase 2	mRNA	0.02
ENSMUSG00000021416|K13239	Eci3	Enoyl-Coenzyme A delta isomerase 3	mRNA	0.27
ENSMUSG00000027674|K13342	Pex5l	Peroxisomal biogenesis factor 5-like	mRNA	0.15
ENSMUSG00000063428|K00272	Ddo	D-aspartate oxidase, isoform CRA_a, partial [Mus musculus]	mRNA	2.35
ENSMUSG00000026272|K00830	Agxt	Serine–pyruvate aminotransferase, mitochondrial isoform 1 precursor [Mus musculus]	mRNA	0.45
ENSMUSG00000027261|K11517	Hao1	Hydroxyacid oxidase 1 [Mus musculus]	mRNA	2.43
ENSMUSG00000046840	Hnf4aos	Hepatic nuclear factor 4 alpha, opposite strand	ncRNA	2.19
ENSMUSG00000048482|K04355	Bdnf	brain derived neurotrophic factor	mRNA	1.26
ENSMUSG00000085667|K00922	Gm12992	predicted gene 12992	mRNA	2.04
ENSMUSG00000020901|K02649	Pik3r5	phosphoinositide-3-kinase regulatory subunit 5	mRNA	0.41
ENSMUSG00000023809|K04373	Rps6ka2	ribosomal protein S6 kinase, polypeptide 2	mRNA	0.47
ENSMUSG00000004933|K08888	Matk	megakaryocyte-associated tyrosine kinase	mRNA	0.08
TNF signaling pathway				
ENSMUSG00000085667|K00922	Gm12992	Predicted gene 12992	ncRNA	2.64
ENSMUSG00000021367|K16366	Edn1	Endothelin 1	mRNA	2.16
ENSMUSG00000035385|K14624	Ccl2	Chemokine (C-C motif) ligand 2	mRNA	2.41
ENSMUSG00000034394|K05419	Lif	Leukemia inhibitory factor	mRNA	3.06
ENSMUSG00000029380|K05505	Cxcl1	Chemokine (C-X-C motif) ligand 1	mRNA	0.20
ENSMUSG00000053113|K04696	Socs3	Suppressor of cytokine signaling 3	mRNA	0.37
ENSMUSG00000021250|K04379	Fos	FBJ osteosarcoma oncogene	mRNA	0.23
ENSMUSG00000027398|K04519	Il1b	Interleukin 1 beta	mRNA	0.31
ENSMUSG00000020901|K02649	Pik3r5	Phosphoinositide-3-kinase regulatory subunit 5	mRNA	0.41
ENSMUSG00000058427|K05505	Cxcl2	Chemokine (C-X-C motif) ligand 2	mRNA	0.04
ENSMUSG00000032487|K11987	Ptgs2	Prostaglandin-endoperoxide synthase 2	mRNA	0.05
Ubiquitin mediated proteolysis				
ENSMUSG00000085995|K10591|K09391	Gm2788	Predicted gene 2788		2.01
ENSMUSG00000105987|K10591	AI506816	Expressed sequence AI506816	ncRNA	2.08
ENSMUSG00000006398|K03363	Cdc20	Cell division cycle 20	mRNA	2.53
ENSMUSG00000053113|K04696	Socs3	Suppressor of cytokine signaling 3	mRNA	0.37
ENSMUSG00000052981|K10582	Ube2ql1	Ubiquitin-conjugating enzyme E2Q family-like 1	mRNA	0.08
ENSMUSG00000111626|K03357	APC10, DOC1	Anaphase-promoting complex subunit 10	mRNA	0.20

Heat shock proteins (Hsp) were increased under various environment stimulus. In this study, we found the expressions of Hsp1, Hspb2 Hsp25-ps1 and Hsp1-like mRNA were significantly upregulated in the diabetic group as compared to the control group. Meanwhile, other inflammation related genes including Interferon regulatory factor 7 (Irf7), cyclin-dependent kinase 1 (Cdk1), cyclin D1. proto-oncogene protein c-fos (FOS), interleukin 1 beta (IL1B), interleukin 1 receptor, type I (IL1r1), monocyte differentiation antigen (CD14), cathepsin K (CTSK), phosphoinositide-3-kinase regulatory subunit 5 (Pik3r5), histocompatibility 2, Q region locus 1 (H2-Q1) were down-regulated. Inflammation is closely associated cell apoptosis, some of these genes were also enriched in cell cycle and apoptosis pathway, such as IL1B.

Peroxisomes are essential organelles exerting key functions in fatty acid metabolism such as the degradation of very long-chain fatty acids. Our results showed that two genes involved in lipid metabolism were significantly up-regulated. Acyl-CoA synthetase long-chain family member 4 (Acsl4) and acyl-CoA synthetase long-chain family member 6 (Acsl6) were isozyme of the long-chain fatty-acid-coenzyme A ligase family. Although differing in substrate specificity, subcellular localization, and tissue distribution, all isozymes of this family converted free long-chain fatty acids into fatty acyl-CoA esters, and thereby played a key role in lipid biosynthesis and fatty acid degradation. ACSL4 has a unique substrate specificity for arachidonic acid and modified membrane lipid composition in a manner favourable to lipid peroxidation. Hepatic ACSL4 is coregulated with the phospholipid (PL)-remodeling enzyme lysophosphatidylcholine (LPC) acyltransferase 3 to modulate the plasma triglyceride (TG) metabolism. Liver-specific knockdown of ACSL4 revealed a substantial decrease in circulating VLDL-TG levels and lipid peroxidation in mice fed a high-fat diet (Singh et al., 2019).

We also found one lipid metabolism related gene was significantly down-regulated. ATP-binding cassette, sub-family D (ALD), member 2 (ABCD2) is a member of the ALD subfamily, which is involved in peroxisomal import of fatty acids and/or fatty acyl-CoAs in the organelle. ABCD2 plays a role in the degradation of long-chain saturated and omega 9-monounsaturated fatty acids and in the synthesis of docosahexanoic acid (DHA) (Fourcade et al., 2009). The absence of ABCD2 altered expression of gene clusters associated with lipid metabolism, including PPAR*α* signaling (Liu et al., 2014). Overexpression of ABCD2 alone prevented oxidative lesions to proteins in a mouse X-linked Adrenoleukodystrophy model (Fourcade et al., 2010) .

Our results showed that the expression of several genes involved in the glucose metabolism were significantly changed (Table S3). Insulin interacts with the insulin receptor, and the activated receptor promotes activity of the phosphoinositide-3 kinase (PI3K) enzyme. The function of differentially expressed lncRNAs were not fully understood, but their predicted target genes such as acetyl-CoA carboxylase beta (Acacb) and fructose bisphosphatase 2 (Fbp2) were involved in the glucose metabolisms.

### qRT-PCR validation of the differentially expressed genes

**Figure 5:**
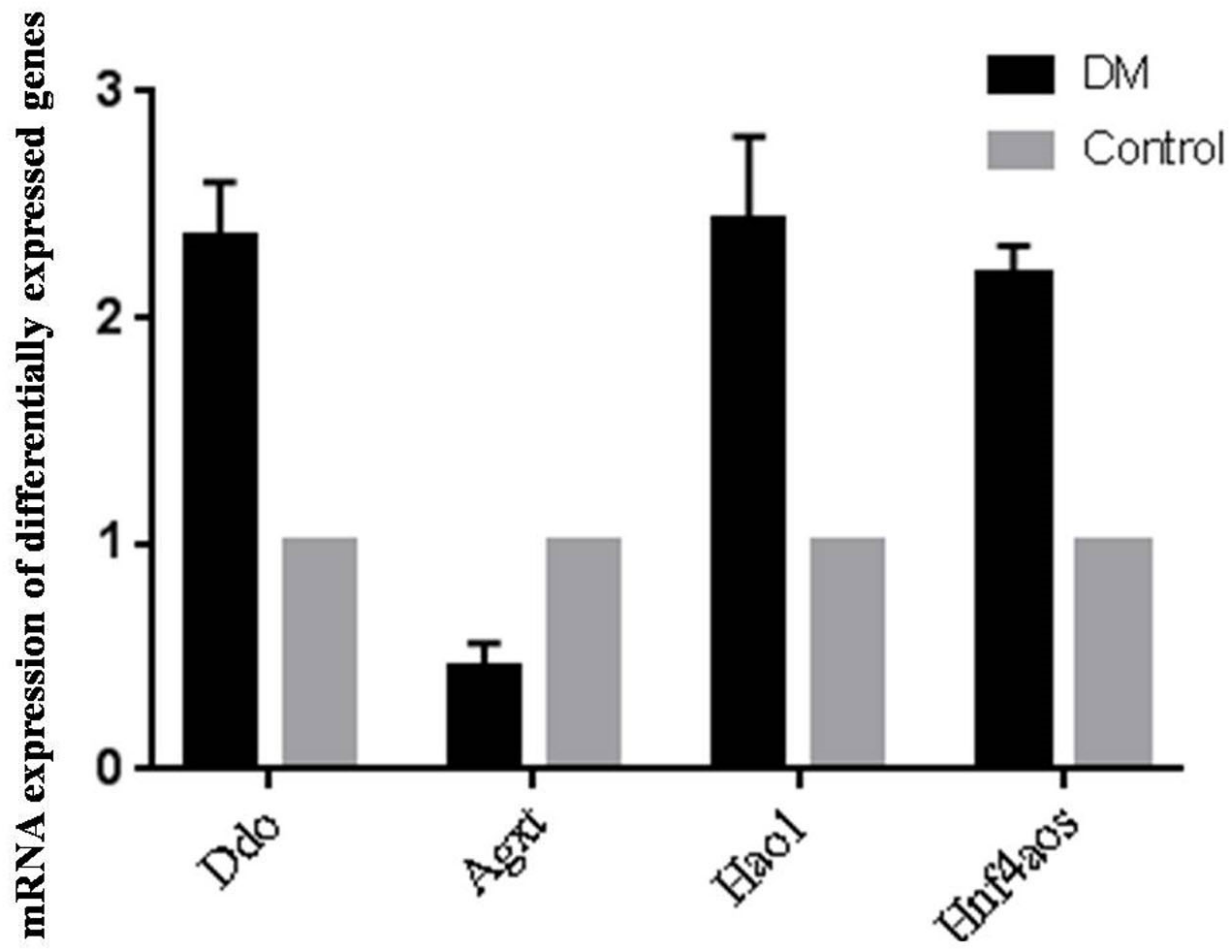
qRT-PCR validations of differentially expressed RNAs.

Since OS is of significance in hepatic metabolism, a detailed inspection of genes involved in peroxosome pathway was chosen for qRT-PCR analysis to validation the RNA-seq data. qRT-PCR primers were designed based on the lncRNA sequences from mapview (https://www.ncbi.nlm.nih.gov/mapview/) (Table S4). The represented DE genes included three mRNAs, D-aspartate oxidase (Ddo), Alanine-glyoxylate aminotransferase (Agxt) and Hydroxyacid oxidase 1(Hao1), and one lncRNA Hnf4aos. qRT-PCR results were shown in Fig. 5. We found the qRT-PCR results were nearly perfect concordance with the RNA-seq results. These findings confirmed the accuracy of microarray data obtained from RNA-seq results.

## Discussion

Diabetes mellitus is characterized by glucose metabolism disorders. More recent studies have found that diabetes also related to OS and ROS intervention (Ceriello & Motz, 2004; Song et al., 2007). Diabetes is known to increase oxidative stress. Previous experimental and clinical data suggests that the generation of ROS increased with diabetes and that the onset of diabetes and its comorbidities and complications are closely associated with oxidative stress (Johansen et al., 2005). High glucose has also been shown to increase oxidative stress (Yildirim et al., 2019), OS parameters were increased and antioxidative parameters were decreased during the oral glucose tolerance test (OGTT). OGTT caused a significantly increase level of SOD and lipid hydroperoxide in the body.

This study confirmed that in the STZ-induced DM mice, the content of lipid peroxidation product MDA and 8-iso-PGF2*α* increased significantly, while the SOD activity decreased significantly. Currently, the best accepted biomarker of oxidative stress is the lipid oxidation product 8-iso-PGF2*α* (Van’t Erve et al., 2016; Van’t Erve et al., 2018). 8-iso-PGF2*α* is formed by a non-enzymatic attack by free radicals on arachidonic acid (a component of lipid cell membranes). The changes in these OS biomarkers concentration indicated that the OS increased in the STZ-induced DM mice. In vitro study has demonstrated that the increase in OS was associated with increased apoptosis of HepG2 cells (Raza & John, 2012).

Adaptation to stress is an essential cellular process. Stress signals trigger a common intracellular signaling cascade, which leads to the activation of the stress-activated protein kinases. In the present study, we identified a series of differentially expressed genes in the livers of STZ-induced diabetic mice upon oxidative stress by RNA sequencing. In total, we found 416 differentially expressed lncRNAs and 910 mRNAs in STZ-diabetic mice compared to control mice. Consistent with previous study (Giannakakis et al., 2015), we also found that dysregulated lncRNAs were associated with negative regulation of transcription from RNA polymerase II promoter in STZ-induced diabetic liver cells. Cellular process enrichment analysis showed the differentially expressed lncRNAs were associated with fatty acid catabolic and oxidation process, protein modification and localization, indicating the potential regulation role of these dysregulated lncRNA in the balance of oxidation and anti-oxidation. Pathway and GO analysis showed that a great number of differentially expressed genes were involved in the inflammation, cell cycle and cell apoptosis, OS and insulin signaling pathway.

The induction of HSP mRNAs indicated the enhanced repair or degradation of proteins damaged by glycoxidation (West, 2000). CTSK is a widely expressed cysteine protease that had enzymatic and non-enzymatic functions in Toll-like receptor signaling pathway. Toll-like receptors sense pathogen-associated molecular patterns and trigger gene-expression changes that ultimately eradicate the invading signaling pathway, such as inflammation, immune regulation, survival and cell proliferation (Lim & Staudt, 2013). IL-1*β* modulates smooth muscle cell phenotype to a distinct inflammatory state via NF- *κ*B-dependent mechanisms (Alexander et al., 2012). IL-1*β* antibody treatment induced a marked reduction in SMC and collagen content in ApoE-/- mice (Gomez et al., 2018). Down-regulation of IL-1*β* and IL-1R1 may reduce the inflammation reaction in liver vessel.

Lipid metabolism related genes changed in a manner favourable to lipid peroxidation. Previous study showed knockdown of ACSL4 decreased circulating VLDL-TG levels and lipid peroxidation in mice fed a high-fat diet. In our study, ACSL4 and ACSL6 were significantly up-regulated in STZ-induced DM mice. ABCD2 plays a role in the degradation of long-chain saturated and omega 9-monounsaturated fatty acids and in the synthesis of docosahexanoic acid. In this study, ABCD2 was significantly down-regulated in STZ-induced DM mice. The expression changes were in accordance with the increase of the content of lipid peroxidation product MDA and 8-iso-PGF2*α*.

Mitochondria and peroxisomes are small ubiquitous organelles. They both play major roles in cell metabolism, especially in terms of fatty acid metabolism, ROS production, and ROS scavenging, and it is now clear that they metabolically interact with each other (Demarquoy & Le Borgne, 2015). Mitochondria are thought to be the primary target of oxidative damage, as ROS was generated mainly as byproducts of mitochondrial respiration. Impaired mitochondrial oxidative phosphorylation was the primary source of ROS (Lucchesi et al., 2013). The ROS further exacerbated lipid peroxidation in the hepatic cell, which eventually led to serious hepatic cell apoptosis and liver damage (Shrilatha & Muralidhara, 2007).

The liver is the main organ of glucose and lipid metabolism, and also is an important place for insulin resistance (Rui, 2014). A single large dose of STZ is used for experiments attempting to cause severe T1DM by direct toxicity to *β* cells. Large doses can cause near total destruction of *β* cells and little insulin production. The oxidative liver damage and apoptosis further affect the binding of insulin to insulin receptor on the liver cell surface, and the insulin signal transduction. Glucose transport and metabolism were regulated by insulin through its signal transduction pathway (Petersen, Vatner & Shulman, 2017). Abnormal insulin signaling pathway can lead to the imbalance of blood glucose (Hatting et al., 2018).

There are some limitations in this study. Firstly, the underlying mechanisms of the dysregulated lncRNAs in pathological of diabetes mellitus were unclear. Secondly, the function and the interaction of these lncRNAs were also unknown. Further studies in how these differentially expressed lncRNAs are involved in the development and progression of diabetic, as well as development of methods to target dysregulated lncRNAs, or evaluate them as biomarkers of early detection of organ dysfunction will be highly needed.

## Conclusions

This study confirmed that the OS was increased in the STZ-induced DM mice as evidenced by the increase of lipid peroxidation product MDA and 8-iso-PGF2*α*. A great number of differentially expressed genes were involved in the inflammation, cell cycle and cell apoptosis, OS and insulin signaling pathway. Although the roles of these RNAs in the metabolism were not fully demonstrated here, these alterations could be used as a foundation for the development of a future investigation of the present RNAs in diabetes.

##  Supplemental Information

10.7717/peerj.8983/supp-1 Figure S1 Characteristic of RNA-sequence data A: Clean reads of RNA sequencing, B Q30 quality distribution, C: Mapping Reads region distribution, D, E: The distribution of gene expression

10.7717/peerj.8983/supp-2 Table S1 Data filtering and Statistical analysis results

10.7717/peerj.8983/supp-3 Table S2 Differentially expressed genes

10.7717/peerj.8983/supp-4 Table S3 The differentially expressed genes enrichment in insulin signal pathway

10.7717/peerj.8983/supp-5 Table S4 qRT-PCR Primers for the differentially expressed RNAs

10.7717/peerj.8983/supp-6 Supplemental Information 1 MIAME checklist

## Abbreviations

STZ: streptozotocin
DM: Diabetes mellitus
lncRNAs: Long noncoding RNAs
GO: Gene Ontology
KEGG: Kyoto Encyclopedia of Genes and Genomes
qRT-PCR: quantitative real-time PCR
lincRNA: long intergenic non-coding RNAs
FPKM: Fragments Per Kilobase of transcript per Million mapped reads
Acacb: acetyl-coenzyme A carboxylase beta
Prkar2b: protein kinase, cAMP dependent regulatory, type II beta
Fbp2: fructose bisphosphatase 2
Ppp1r3d: protein phosphatase 1, regulatory subunit 3D

## Additional Information and Declarations

### Data Availability

The following information was supplied regarding data availability: The raw data is available at NCBI: PRJNA562053.
