# Supplementary figures and images for: Changes of liver transcriptome profiles following oxidative stress in streptozotocin-induced diabetes in mice
Source: PeerJ. 2020 May 27;8:e8983. doi: 10.7717/peerj.8983 (PMC7261117; doi:10.7717/peerj.8983)

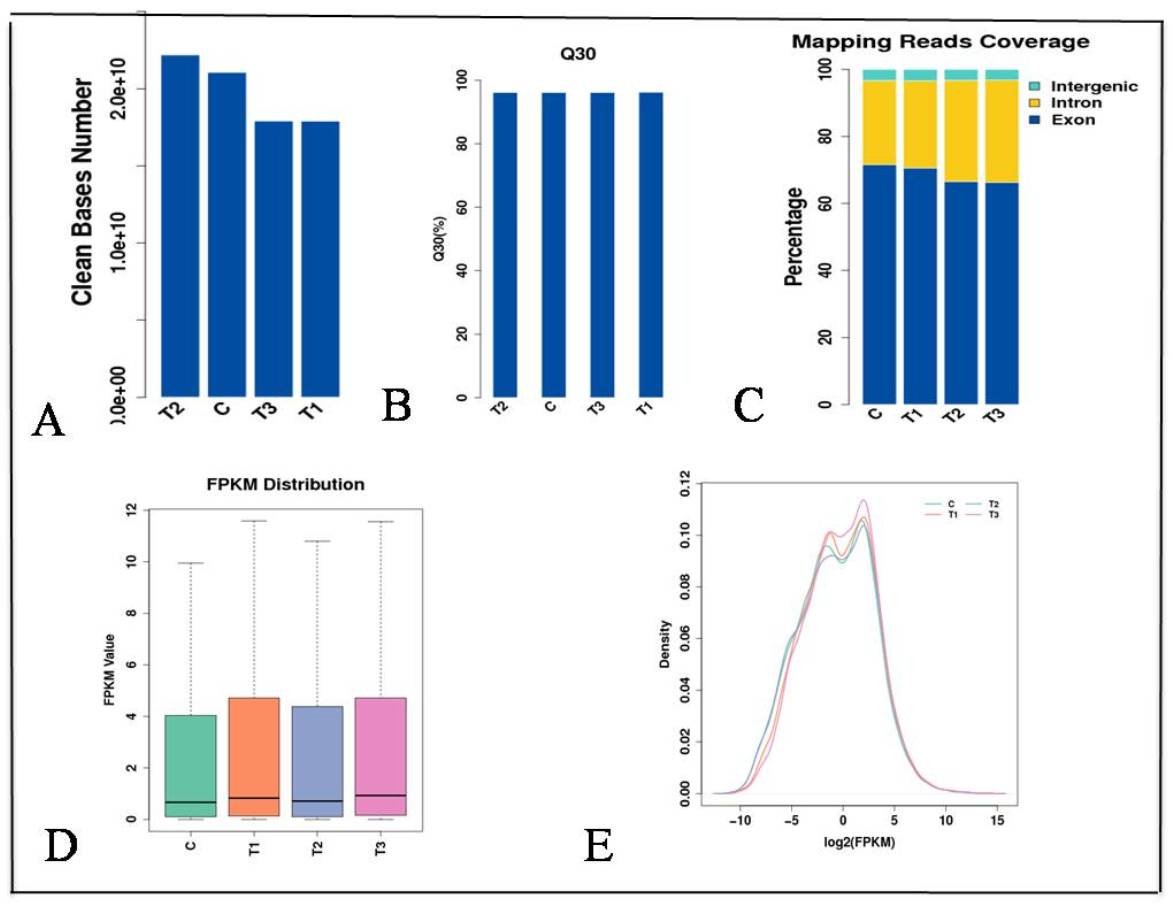

Supplement: Figure S1 — A: Clean reads of RNA sequencing, B Q30 quality distribution, C: Mapping Reads region distribution, D, E: The distribution of gene expression [file peerj-08-8983-s001.jpg]
