# Supplementary material for: Changes of liver transcriptome profiles following oxidative stress in streptozotocin-induced diabetes in mice
Source: PeerJ. 2020 May 27;8:e8983. doi: 10.7717/peerj.8983 (PMC7261117; doi:10.7717/peerj.8983)
Supplement: Table S1 [file peerj-08-8983-s002.docx]

Supplement table 1 Data filtering and Statistical analysis results

| Sample ID | C | T1 | T2 | T3 |
| --- | --- | --- | --- | --- |
| Raw Reads Number | 161,163,054 | 136,948,824 | 171,261,902 | 138,095,052 |
| Raw Bases Number | 24,174,458,100 | 20,542,323,600 | 25,689,285,300 | 20,714,257,800 |
| Clean Reads Number | 140,608,350 | 119,439,990 | 148,242,974 | 119,534,452 |
| Clean Reads Rate(%) | 87.25 | 87.22 | 86.56 | 86.56 |
| Clean Bases Number | 21,091,252,500 | 17,915,998,500 | 22,236,446,100 | 17,930,167,800 |
| Low-quality Reads Number | 18,780,944 | 15,754,062 | 20,742,354 | 16,856,102 |
| Low-quality Reads Rate(%) | 11.65 | 11.5 | 12.11 | 12.21 |
| Adapter polluted Reads Number | 1,592,726 | 1,642,650 | 1,977,964 | 1,577,946 |
| Adapter polluted Reads Rate(%) | 0.99 | 1.2 | 1.16 | 1.14 |
| Raw Q30 Bases Rate(%) | 92.39 | 92.5 | 92.23 | 92.15 |
| Clean Q30 Bases Rate(%) | 96.1 | 96.21 | 96.16 | 96.1 |
| rRNA Mapping Reads Number | 181,034 | 112,122 | 298,610 | 126,552 |
| rRNA Mapping Rate(%) | 0.11 | 0.08 | 0.17 | 0.09 |
| Total Clean Reads Number | 140,427,316 | 119,327,868 | 147,944,364 | 119,407,900 |
| Total Clean Bases Number | 21,064,097,400 | 17,899,180,200 | 22,191,654,600 | 17,911,185,000 |
| Total Q30(%) | 95.99 | 96.13 | 95.99 | 96.01 |
