# Supplementary material for: Changes of liver transcriptome profiles following oxidative stress in streptozotocin-induced diabetes in mice
Source: PeerJ. 2020 May 27;8:e8983. doi: 10.7717/peerj.8983 (PMC7261117; doi:10.7717/peerj.8983)
Supplement: Table S3 [file peerj-08-8983-s004.docx]

| LncRNA | DE Gene name | Correlation | C | T1 | T2 | T3 | predicted trans-target Gene | Biotype | Position |  |  |
| --- | --- | --- | --- | --- | --- | --- | --- | --- | --- | --- | --- |
| MSTRG.65096.1 | ENSMUSG00000049999 | -0.9487 | 27.88809 | 71.67934 | 35.80746 | 32.71174 | Ppp1r3d | linc | chr2:75637992-75644273:+ | | |
| ENSMUSG00000105260 | ENSMUSG00000082926 | 1 | 1.229773 | 4.332112 | 2.111656 | 2.084824 | Gm8172 | lincRNA | chr3:41477577-41482391:- | | |
| MSTRG.59891.1 | ENSMUSG00000042010 | 1 | 0.821129 | 7.331541 | 9.461131 | 2.074081 | Acacb | antisense | chr19:37676943-37697800:- | | |
| MSTRG.77081.1 | ENSMUSG00000021456 | -1 | 0.749236 | 2.955938 | 1.671271 | 3.231195 | Fbp2 | linc | chr4:3187112-3236548:- | | |
| ENSMUSG00000097768 | ENSMUSG00000002997 | -1 | 0.28835 | 1.79957 | 0.362358 | 3.826313 | Prkar2b | lincRNA | chr16:93792150-93794888:- | | |
| MSTRG.52981 | ENSMUSG00000020901 | 1 | 0.39202 | 0 | 0.087757 | 0.735144 | Pik3r5 | antisense | chr17:78947599-78948322:- | | |
| MSTRG.107691 | ENSMUSG00000081121 | 1 | 1.691103 | 3.433158 | 0.414787 | 3.244604 | Gm12791 | linc | chr8:3046915-3049637:+ | | |
| MSTRG.83762 | ENSMUSG00000085667 | 1 | 0.457279 | 1.087811 | 0.743708 | 1.029825 | Gm12992 | linc | chr4:139331217-139337456:- | | |
| MSTRG.52421 | ENSMUSG00000042717 | -1 | 0.251112 | 0.036216 | 0.272251 | 0.360103 | Ppp1r3a | antisense | chr17:65885163-65887311:+ | | |
| MSTRG.118294 | ENSMUSG00000078650 | -1 | 0.502491 | 1.955562 | 0.092846 | 2.275001 | G6pc | linc | chr9:95581478-95594070:+ | | |
| ENSMUSG00000085439 | ENSMUSG00000083909 | 1 | 0.207377 | 1.74045 | 2.460432 | 0.486111 | Gm15842 | antisense | chr2:72097603-72148796:- | | |
| MSTRG.42358 | ENSMUSG00000046709 | 1 | 0.133073 | 0.564443 | 0.797766 | 0.207624 | Mapk10 | antisense | chr15:76118138-76122271:+ | | |
| MSTRG.72458 | ENSMUSG00000025537 | 1 | 0.214262 | 0.443243 | 0.135471 | 0.827815 | Phkg1 | antisense | chr3:58108059-58119002:- | | |
| MSTRG.65122 | ENSMUSG00000083045 | -1 | 0.262964 | 0.57939 | 0.241301 | 0.129947 | Gm16100 | linc | chr2:75786028-75788936:+ | | |
| ENSMUSG00000110386 | ENSMUSG00000029167 | -1 | 0.021597 | 1.926078 | 0.005237 | 3.172138 | Ppargc1a | lincRNA | chr8:85125872-85132289:- | | |
| ENSMUSG00000084923 | ENSMUSG00000053113 | -1 | 16.5446 | 4.332966 | 0.607923 | 18.42501 | Socs3 | lincRNA | chr5:8998402-8999669:- | | |
| ENSMUSG00000108431 | ENSMUSG00000041798 | -1 | 1.10654 | 0.083735 | 1.325191 | 0.149866 | Gck | antisense | chr7:26304421-26307286:- | | |

Supplement table 4 The differentially expressed genes enrichment in insulin signal pathway
