# Supplementary material for: Changes of liver transcriptome profiles following oxidative stress in streptozotocin-induced diabetes in mice
Source: PeerJ. 2020 May 27;8:e8983. doi: 10.7717/peerj.8983 (PMC7261117; doi:10.7717/peerj.8983)
Supplement: Table S4 [file peerj-08-8983-s005.docx]

| Table 2 qRT-PCR Primers for the differentially expressed RNAs | | |  |  |
| --- | --- | --- | --- | --- |
| **LncRNA** | **Gene name** | **Primer sequence (5'->3')** | | **Product length** |
| ENSMUSG00000027674 | Pex5l | F：GCGTCCTGTAGCCCGTAG | | 140 |
|  |  | R：CCCTGGTACATTCTGCTCCG | |  |
| ENSMUSG00000027261 | Hao1 | F：TCTATCCACGGATGCTTCGC | | 92 |
|  |  | R：AGCCCCAACACATATTGGCA | |  |
| ENSMUSG00000063428 | Ddo | F：ACAGTGTGTATTGCGGTCGT  R：AAAAGCAAAGGCTGCACACG | | 177 |
| ENSMUSG00000026272 | Agxt | F：TGGAGGGACATCGTCAGCTA | | 90 |
|  |  | R：AATCCGTAGCACCCTCTCCT | |  |
| ENSMUSG00000027261 | Hnf4aos | F：CTATCCACGGATGCTTCGCA | | 91 |
|  |  | R：AGCCCCAACACATATTGGCA | |  |
| β-actin |  | F：AGTGTGACGTTGACATCCGTA | | 112 |
|  |  | R：CCAGAGCAGTAATCTCCTTCT | |  |
