# Supplementary material for: Changes of liver transcriptome profiles following oxidative stress in streptozotocin-induced diabetes in mice
Source: PeerJ. 2020 May 27;8:e8983. doi: 10.7717/peerj.8983 (PMC7261117; doi:10.7717/peerj.8983)
Supplement: Supplemental Information 1 [file peerj-08-8983-s006.docx]

MIAME checklist

1. Experiment description

SAMPLE: dzsgz6 (SAMN12635940)

EXPERIMENT: Control (SRX7095329) pooled control sample

RUN: dzsgz6_1.fq.gz (SRR10394954)

EXPERIMENT: Diabetic group (SRX7095330)

RUN: tnbsgz2_1.fq.gz (SRR10394953) three biological replicates

1. Array design

3 μg of total RNA was used for sequencing preparation using NEB Next Ultra Directional RNA LibraryPrep Kit for Illumina (NEB，Ispawich，USA) kit along with Ribo-Zero Gold rRNA (Illumina Inc., CA, USA) to remove rRNA. The resulting libraries were sequenced on a HiSeq 2000 (Illumina Inc., CA, USA) instrument that generated paired-end reads of 100 nucleotides.

1. Samples

Specific pathogen-free male C57BL/6 mice weighing 20-22g were injected with citrate buffer as normal control group. RNA extracted from the liver tissues of three control mice were pooled together as control group for sequencing.

Specific pathogen-free male C57BL/6 mice weighing 20-22g received one intra-peritoneal injection of 130mg/kg streptozotocin (STZ, Sigma, St. Louis, MO, USA) solution in 0.05 M citrate buffer (pH 4.5) to induce diabetes. Blood glucose (BG) was measured to confirm diabetes, which was defined as glycemia higher than 16.7mmol/L. RNA extracted from the liver tissues of STZ-induced diabetic mice (n=3) were sequencing separately as DM groups.

1. Hybridizations

The resulting libraries were sequenced on a HiSeq 2000 (Illumina Inc., CA, USA) instrument that generated paired-end reads of 100 nucleotides. The reference genomes and the annotation file were downloaded from ENSEMBL database (http://www.ensembl.org/index.html). And Clean Data were mapped to the reference genome using HISAT2 (<http://ccb.jhu.edu/software/hisat2>/index. shtml).

1. Measurements

The liver transcriptome was reconstructed from all of the RNA-seq datasets using StringTie 1.3.2.d (http://ccb.jhu.edu/software/stringtie/). DESeq (<http://www>. bioconductor.org/packages/release/bioc/html/DESeq. html) was used for differential expression analysis between diabetic and normal mice liver transcriptomes. Differentially expressed genes were identified based on threshold changes of ≥2-fold or ≤ -2-fold and q values ≤ 0.05. The data were normalized and hierarchically clustered with R software 3.1.1.

1. Normalization controls

Raw Data are processed with Perl scripts to ensure the quality of data used in following analysis. The adopted filter criteria are:

1) Filter out adaptor-polluted reads. (Reads contains more than 5 adapter-polluted bases are regarded as adaptor-polluted reads and are filtered out. As for paired-end sequencing data, reads from two ends are filtered out if any read of the paired-end reads are adaptor-polluted);

2) Filter out low-quality reads. (Reads with the number of bases whose phred Quality value was no more than 19 accounting for more than 15% are regarded as low-quality reads and are filtered out.)

3) Filter out reads with the number of N basesaccounting for more than 5 %.

As for paired-end sequencing data, reads from two ends are filtered out if any read of the paired-end reads should be filtered out according to the abovecriteria.

The Clean Data is abotained after filtering, and statistics analyses are performed on its quantity and quality, including Q30 statistics, data quantity statistics, base content statistics, etc.
